# Monitoring time domain characteristics of Parkinson’s disease using 3D memristive neuromorphic system

**DOI:** 10.3389/fncom.2023.1274575

**Published:** 2023-12-15

**Authors:** Md Abu Bakr Siddique, Yan Zhang, Hongyu An

**Affiliations:** ^1^Department of Electrical and Computer Engineering, Michigan Technological University, Houghton, MI, United States; ^2^Department of Biological Sciences, Michigan Technological University, Houghton, MI, United States

**Keywords:** memristors, neuromorphic computing, spiking neural networks, deep brain stimulation, Parkinson’s disease

## Abstract

**Introduction:**

Parkinson’s disease (PD) is a neurodegenerative disorder affecting millions of patients. Closed-Loop Deep Brain Stimulation (CL-DBS) is a therapy that can alleviate the symptoms of PD. The CL-DBS system consists of an electrode sending electrical stimulation signals to a specific region of the brain and a battery-powered stimulator implanted in the chest. The electrical stimuli in CL-DBS systems need to be adjusted in real-time in accordance with the state of PD symptoms. Therefore, fast and precise monitoring of PD symptoms is a critical function for CL-DBS systems. However, the current CL-DBS techniques suffer from high computational demands for real-time PD symptom monitoring, which are not feasible for implanted and wearable medical devices.

**Methods:**

In this paper, we present an energy-efficient neuromorphic PD symptom detector using memristive three-dimensional integrated circuits (3D-ICs). The excessive oscillation at beta frequencies (13–35 Hz) at the subthalamic nucleus (STN) is used as a biomarker of PD symptoms.

**Results:**

Simulation results demonstrate that our neuromorphic PD detector, implemented with an 8-layer spiking Long Short-Term Memory (S-LSTM), excels in recognizing PD symptoms, achieving a training accuracy of 99.74% and a validation accuracy of 99.52% for a 75%–25% data split. Furthermore, we evaluated the improvement of our neuromorphic CL-DBS detector using NeuroSIM. The chip area, latency, energy, and power consumption of our CL-DBS detector were reduced by 47.4%, 66.63%, 65.6%, and 67.5%, respectively, for monolithic 3D-ICs. Similarly, for heterogeneous 3D-ICs, employing memristive synapses to replace traditional Static Random Access Memory (SRAM) resulted in reductions of 44.8%, 64.75%, 65.28%, and 67.7% in chip area, latency, and power usage.

**Discussion:**

This study introduces a novel approach for PD symptom evaluation by directly utilizing spiking signals from neural activities in the time domain. This method significantly reduces the time and energy required for signal conversion compared to traditional frequency domain approaches. The study pioneers the use of neuromorphic computing and memristors in designing CL-DBS systems, surpassing SRAM-based designs in chip design area, latency, and energy efficiency. Lastly, the proposed neuromorphic PD detector demonstrates high resilience to timing variations in brain neural signals, as confirmed by robustness analysis.

## 1 Introduction

Parkinson’s disease (PD) is a prevalent neurodegenerative condition affecting millions of patients worldwide (Ghasemi et al., 2018; Zhou et al., 2018). Although various medications are available to alleviate the symptoms, their effectiveness tends to diminish over time due to drug resistance. Consequently, later stages of PD patients require higher medication dosages, which can significantly impact cognitive abilities and mental health (Dostrovsky and Lozano, 2002; Arlotti et al., 2016). To address this challenge, deep brain stimulation (DBS) has emerged as a novel therapy for PD patients in advanced stages. In a DBS system, an electrode is implanted into a specific target in the brain for delivering electrical stimulation signals through a battery-powered programmable stimulator implanted in the chest of PD patients. The current DBS system continuously sends the stimulation signals to the brain with fixed parameters and frequency regardless of the clinical state of the patient, referred to as an open-loop DBS (OL-DBS) system (Ghasemi et al., 2018; Zhou et al., 2018; Lozano et al., 2019). The rigid fashion of the current OL-DBS technique poses two critical issues: (1) the high-frequency stimulation causes serious cognitive and psychiatric side effects, such as speech deficits and cognitive dysfunction (Dostrovsky and Lozano, 2002; Deuschl et al., 2006; Massano and Garrett, 2012; Arlotti et al., 2016; Cyron, 2016); (2) the continuous stimulation quickly drains the battery of energy-inefficient hardware platforms (Salam et al., 2015; Shukla, 2015; Ghasemi et al., 2018; Jovanov et al., 2018; Shah et al., 2018; Zhou et al., 2018). Therefore, a closed-loop DBS (CL-DBS) system (He et al., 2021) has been proposed to address the limitations of the OL-DBS system by incorporating a feedback loop. This feedback loop allows the detection of PD symptoms and the delivery of optimized stimulus impulses according to different severities of PD symptoms. The CL-DBS systems are widely identified as the future development direction of the DBS system (Allen et al., 2010; Rosin et al., 2011; Carron et al., 2013; Shukla, 2015; Arlotti et al., 2016; Little et al., 2016; Rossi et al., 2016; Ghasemi et al., 2018; Kuo et al., 2018; Zhou et al., 2018; Lozano et al., 2019; Velisar et al., 2019). In a CL-DBS system, stimulation parameters are automatically adjusted based on the clinical symptoms of the PD patients. The studies demonstrate that closed-loop paradigms with real-time adaptive stimulation yield fewer unpleasant side effects and greater clinical benefits compared to fixed paradigms (He et al., 2021; Su et al., 2021). CL-DBS systems that implement simple on-off control of stimulations have been developed and tested in human and animal studies (Marceglia et al., 2007; Little et al., 2013; Priori et al., 2013; Wu et al., 2015; He et al., 2021).

There are various challenges associated with CL-DBS systems. The continuous operation of implanted CL-DBS systems round-the-clock, 7 days a week, poses significant demands in terms of intelligence and energy efficiency. Accurately recognizing symptom-related signals and generating adaptive stimulation signals usually require computationally expensive algorithms, e.g., reinforcement learning (Shukla, 2015; Arlotti et al., 2016; Little et al., 2016; Kuo et al., 2018; Lozano et al., 2019; Velisar et al., 2019; Gao et al., 2020; Liu et al., 2020). Thus, a novel design of an intelligent CL-DBS device with low power consumption and high intelligence is in urgent demand. In this work, we apply a three-dimensional (3D) memristive neuromorphic system to the energy-efficient recognition and assessment of symptoms in a CL-DBS system. Specifically, we utilize the PD computational model (Kumaravelu et al., 2016), which includes a cortical-basal ganglion-thalamic circuit, to generate a substantial amount of data from the healthy and Parkinsonian rat brain. The Parkinson’s symptom is identified with the output of this PD computational model at the beta frequency range (13–30 Hz). The heightened power density of neural activities in the beta frequency range has been positively correlated with the severity of motor impairment (Perez-Alcazar et al., 2010; Connolly et al., 2015; de Hemptinne et al., 2015; Escobar et al., 2017). Therefore, the power density levels in the beta frequency range can serve as biomarkers for evaluating PD symptoms. The generated data with the PD computational models are used for training a novel neuromorphic PD detector that is implemented with a spiking long-short-term memory neural network (S-LSTM) (An et al., 2018a,b). The neuromorphic PD detector will be trained using the Whetstone method (Severa et al., 2019). The Whetstone method is a cutting-edge training algorithm for neuromorphic systems that gradually transforms conventional nonlinear functions, e.g., sigmoid function, into threshold functions during the training process. Furthermore, our neuromorphic PD detector can identify PD symptoms based on neural activities in time domain without converting them into the frequency domain, resulting in enhanced computational efficiency. The incorporation of memristive synapses in our neuromorphic PD detector significantly improves energy efficiency, a crucial factor for CL-DBS systems. The energy efficiency of our neuromorphic CL-DBS system is evaluated using a hardware simulator, named NeuroSIM (Chen et al., 2018; Peng et al., 2020; Lu et al., 2021; An et al., 2021a,b). Specifically, the weights and biases of the neuromorphic PD detector are saved and deployed into the NeuroSIM simulator as memristive synapses. After that, the hardware performance of our neuromorphic PD detector will be calculated under both monolithic and heterogeneous 3D chip architectures.

The contributions of this study are outlined as follows:

1. Utilizing spiking signals from the neural activities directly in time domain for PD symptom evaluation significantly reduces the time and energy required for converting signals from the time domain to the frequency domain.
2. To our best knowledge, we are the first to employ neuromorphic computing and memristors in the design of CL-DBS systems.
3. The neuromorphic PD detector with memristive synapse architectures outperforms traditional SRAM-based designs in CL-DBS systems regarding chip design area, latency, and energy efficiency.
4. Our study evaluates the enhancements gained by implementing three-dimensional technology in hardware for CL-DBS, considering chip design area, latency, and energy efficiency.
5. The robustness analysis of our neuromorphic PD detector demonstrates its high resilience to timing variations in brain neural signals.

## 2 Research background

### 2.1 Introduction to neuromorphic computing

The brains can perform remarkably intricate tasks with incredible energy efficiency. The adult human brain consumes approximately 20 W of power consumption (Jorgensen, 2021). In contrast, the average energy usage of modern digital computers is about 60–175 watts (Marković et al., 2020; Jorgensen, 2021) for comparable cognitive tasks. For instance, a typical computer requires approximately 250 watts of power to recognize just 1,000 unique items (Roy et al., 2019). Training a sophisticated natural language processing model on a modern supercomputer consumes 1,000 kWh of energy, equivalent to the energy needs of a human brain performing all its tasks for 6 years (Marković et al., 2020). The remarkable outperformance of the human brain can be attributed to several fundamental features, including the extensive high density of connectivity, spike-based information representation, and a structural and functional hierarchical organization (Felleman and Van Essen, 1991; Bullmore and Sporns, 2012). The human brain is estimated to have over 100 billion neurons connecting with trillions of synapses (Changeux, 1997). Synapses serve as the storage components for memory and learning, while neurons act as the computational units of the brain, exchanging information through discrete action potentials or spikes. Neuromorphic computing is a novel computational paradigm that seeks to replicate the functionality of biological neurons and synapses of the brains. The concept of neuromorphic computing was first envisioned by Mead in the 1980s (Mead and Ismail, 1989; An et al., 2018a,b).

The primary objective of neuromorphic computing is to create brain-inspired computations that overcome the challenges from the traditional von Neumann computer architecture (Davies et al., 2021). The von Neumann architecture consists of separate memory units and central processing units (CPU). Consequently, information must be repeatedly transferred between these units during computations, leading to speed bottlenecks and limitations in energy efficiency, widely known as the von Neumann bottleneck (Naylor and Runciman, 2007; Min and Corinto, 2021). Neuromorphic computing encompasses various technologies to overcome the von Neumann bottleneck (Mead, 1990). With co-located electronic neurons and synapses, neuromorphic chips, such as Intel Loihi, provide a much faster and energy-efficient computational paradigm (Wunderlich et al., 2019).

Additionally, the remarkable energy efficiency of neuromorphic systems can be attributed to the distinctive information coding schemes found in biological neural systems (Roy et al., 2019). In neural systems, the communication information among neurons is coded in a sequence of spiking signals at low frequency. Unlike the high-speed modern computer, the main frequency of the spiking signals in the nervous system is as low as ~kilohertz level (1–10 millisecond duration) with millivolt-level magnitudes (Kandel et al., 2000). The neuromorphic system is a software and hardware co-design approach to achieving a comparable power-efficient artificial intelligence system by taking inspiration from human brains and implementing low-fire-rate spiking communication, threshold activation functions, and spiking neural networks (Mead, 1990; Schemmel et al., 2008; Azevedo et al., 2009; Gerstner and Naud, 2009; De Garis et al., 2010; Goertzel et al., 2010; Smith, 2010; Versace and Chandler, 2010; Brüderle et al., 2011; Merolla et al., 2011; Seo et al., 2011; Joubert et al., 2012; Pfeil et al., 2012; Esser et al., 2013; Furber et al., 2013; Hasler and Marr, 2013; Painkras et al., 2013; Rajendran et al., 2013; Stromatias et al., 2013; Benjamin et al., 2014; Chen et al., 2014; Merolla et al., 2014; Putnam et al., 2014; Indiveri et al., 2015; Qiao et al., 2015; Schuman et al., 2015; Walter et al., 2015; Schuman, 2016; Ehsan et al., 2017; Ferreira de Lima et al., 2017; Lastras-Montaño et al., 2017; Osswald et al., 2017; Schuman et al., 2017; Bai and Bradley, 2018; Davies et al., 2018; An et al., 2018a,b; Severa et al., 2019). In contrast to conventional artificial neural networks, e.g., convolution neural networks (LeCun et al., 1989; Lecun et al., 2015; Bengio et al., 2017), recurrent neural networks (Zaremba et al., 2014; Bengio et al., 2017), etc., the information conveyed between layers in SNNs and neuromorphic systems is in a format of spiking pulses (Zenke and Ganguli, 2018; Neftci et al., 2019; Tavanaei et al., 2019; Taherkhani et al., 2020). Through this way, SNNs have the capability of emulating the functionalities and learning processes of biological neural networks (Maass, 1997). In an SNN, neurons interact through spikes transmitted via adjustable synapses (An et al., 2021a,b). While neurons in traditional ANNs exhibit fixed continuous-valued activity, biological neurons employ discrete spikes to compute and transmit information. The sparsity of low-frequency neuron spikes significantly increase the energy efficiency of the neuromorphic system (Yi et al., 2015). SNNs utilize biological neuron models for computation, bridging the gap between neuroscience and AI (Yamazaki et al., 2022). Furthermore, the activation functions, which are also referred to as neurons in deep learning, are threshold activation functions rather than traditional nonlinear activation functions in SNNs (Ramachandran et al., 2017; Lau and Lim, 2018; Nwankpa et al., 2018). Thus, the outputs of the threshold functions are either zero or one, which decreases the computational complexity of algorithms and hardware implementations.

The discrete spiking signals require particular training algorithms and encoding paradigms (Roy et al., 2019; Mead, 2020). In a digital system, the analog signals will be converted into binary numbers using analog-to-digital converters (ADCs) for further Boolean calculations (Indiveri et al., 2015; Liu et al., 2015; An, 2020). But in brains, the exterior analog signals, such as visual and auditory signals captured by sensory organs, are converted into a sequence of spikes (Kandel et al., 2000; Gerstner et al., 2012). Thus, the communication among neurons is the spiking signals. Several encoding methods are available, e.g., temporal encoding (Sakemi et al., 2020), rate encoding (Liu et al., 2009; Liu and Delbruck, 2010; Plank et al., 2018), and spatial-temporal encoding (Jin et al., 2018). Several training methods for SNNs have been proposed, including converting traditional ANNs into an SNN after the training process (O’Connor et al., 2013; Diehl et al., 2015; Esser et al., 2015, 2016; Rueckauer et al., 2017; Shrestha and Orchard, 2018; Severa et al., 2019), using biologically plausible algorithms, e.g., spike-timing-dependent plasticity (STDP), to directly train SNN from the beginning (Bohte et al., 2002; Shrestha and Song, 2017), or utilizing an approximation method for mimicking backpropagation training methods (Lee et al., 2016; Panda and Roy, 2016; Zenke and Ganguli, 2018), e.g., SpikeProp (Bohte et al., 2002; McKennoch et al., 2006; Shrestha and Song, 2017). These training methodologies particularly designed for SNNs and neuromorphic systems already have competitive training/inference accuracies (Wade et al., 2008; Lee et al., 2016; Severa et al., 2019) compared to conventional deep learning (O’Connor et al., 2013; Diehl et al., 2015; Esser et al., 2015, 2016; Guo et al., 2017; Rueckauer et al., 2017; Yan et al., 2018). Numerous neuromorphic chips are launched to further enhance the capability of neuromorphic computing, such as the Loihi chip (Davies et al., 2018), TrueNorth (Akopyan et al., 2015), etc. The Loihi chips are a digital-analog mix specially designed for adaptive self-modifying event-driven fine-grained parallel computations used to implement learning and inference with high power efficiency. The Loihi chip incorporates 128 neuromorphic cores fabricated on Intel’s 14 nm process and features a unique programmable microcode learning engine for on-chip SNN training. The power consumption of Loihi chips is significantly lower (109 X) than other state-of-the-art computing platforms, such as field-programmable gate array (FPGA), central processing unit (CPU), and graphics processing units (GPUs) (Lecun et al., 2015; Goodfellow et al., 2016; Schuman et al., 2017; Blouw et al., 2019; Roy et al., 2019). The distinctive capabilities and high energy efficiency of neuromorphic systems and SNNs offer invaluable advantages to CL-DBS systems and other implantable/wearable medical devices that demand low latency and energy efficiency.

### 2.2 Introduction to deep brain stimulation for Parkinson’s disease

The DBS technique is a neurosurgical procedure that implants special electrodes in specific regions of the brain for sending electrical stimulations. The DBS system consists of two essential components: the electrodes implanted in the brain and a stimulation generator placed in the chest. The patients would be carrying the entire DBS device all the time. The electrode is typically implanted into a specific region of the brain through a small hole in the skull. A thin wire connects the electrode to an implantable pulse generator. The pulse generator serves as the source of electrical stimulation. The DBS system is widely used for neurological diseases, such as Parkinson’s disease, dystonia, and Alzheimer’s Disease (Fang and Tolleson, 2017; Ghasemi et al., 2018; Zhou et al., 2018; Lozano et al., 2019).

**Figure 1:**
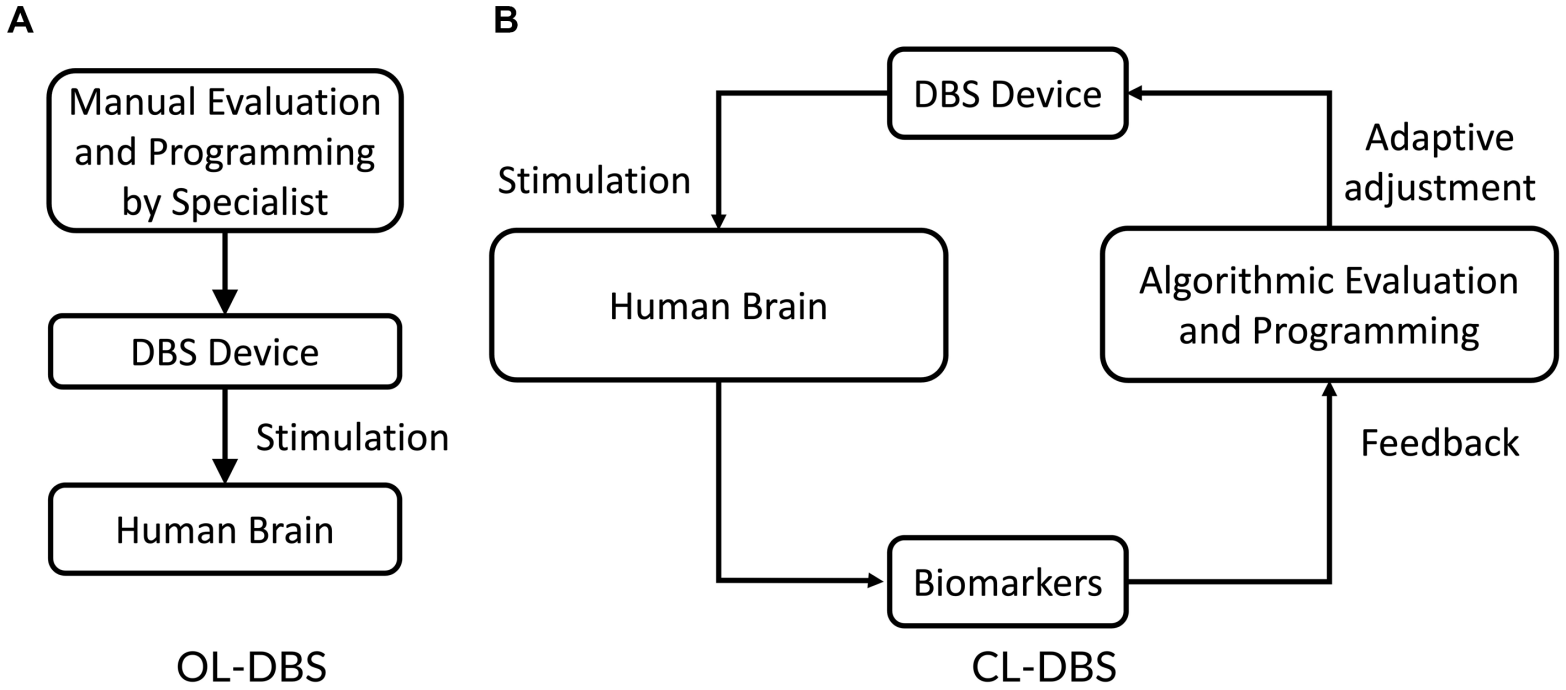
Illustration of open-loop **(A)** and closed-loop **(B)** DBS systems.

Parkinson’s disease is a multifaceted neurodegenerative disorder primarily characterized by the degeneration of dopamine-producing neurons in the brain, resulting in a wide array of motor symptoms. The symptoms of Parkinson’s disease include tremors, bradykinesia, stiffness, abnormal walking, etc. While medications are available to manage certain symptoms, they cannot halt or reverse the underlying neurodegenerative process. Thus, Parkinson’s disease is a complex condition, and treatment plans may involve a combination of medications, physical therapy, and lifestyle modifications. Researchers continue to investigate new therapeutic approaches and potential interventions to slow the progression of the disease and improve the quality of life for patients. Levodopa, a precursor to dopamine, is a frequently prescribed medication that aids in replenishing dopamine levels in the brain and can alleviate motor symptoms (Fahn et al., 2004). However, long-term use of levodopa often results in a condition known as “levodopa-induced dyskinesia,” which is characterized by uncontrollable and writhing movements (Fang and Tolleson, 2017). Moreover, as the disease progresses and the number of dopamine-producing neurons continues to decline, the effectiveness of these medications diminishes over time. In addition, the current medications also have side effects, such as cognitive decline, sleep disturbances, and mood disorders, which significantly impact the patient’s quality of life. When medications are no longer able to provide an adequate quality of life, DBS treatment is considered. Clinical trials have provided evidence for the efficacy of regular electrical stimulation of specific brain regions, such as the subthalamic nucleus (STN), in mitigating the symptoms of Parkinson’s disease. The stimulation frequency of a typical DBS system is commonly classified into two categories: high frequency (typically above 100 Hz, such as 130 or 150 Hz) and low frequency (typically below 100 Hz, such as 60 or 80 Hz) (Su et al., 2018). The therapeutic outcomes on motor function in individuals with Parkinson’s disease (PD) can differ substantially depending on the selected stimulation frequency. Low stimulation frequencies have demonstrated no significant impact on motor symptoms, whereas high stimulation frequencies have shown therapeutic benefits to the patients. Continuous electrical stimulation of brain targets such as STN and GPi has been shown to relieve the symptoms of movement disorders of Parkinson’s disease. This conventional DBS system is referred to as an open-loop DBS (OL-DBS) system (Figure 1A). However, high-frequency stimulation may induce unexpected cognitive and psychiatric side effects such as depression and speech disorders (Dostrovsky and Lozano, 2002; Hariz et al., 2008; Hwynn et al., 2011; Arlotti et al., 2016; Alomar et al., 2017). Additionally, it has the potential to exacerbate axial symptoms and manifestations that often arise during the long-term progression of the disease and treatment, including challenges with gait, speech, and swallowing (di Biase and Fasano, 2016). Another challenge related to OL-DBS system is the high energy consumption associated with continuous stimulation, leading to rapid depletion of the power in implanted devices. Consequently, patients often require additional surgical procedures to replace the neurostimulator battery. Another challenge of the OL-DBS system is the diversity and variability of individual patients. This variability necessitates personalized approaches to OL-DBS system and requires considering the unique characteristics of stimulation signals of each patient. To overcome these limitations, a novel closed-loop DBS (CL-DBS) system is proposed, which incorporates a feedback loop as illustrated in Figure 1B.

The essential distinction between CL-DBS and OL-DBS systems lies in their approach to monitoring PD symptoms and adjusting stimulation parameters accordingly. In an OL-DBS system, the stimulation parameters, e.g., frequency, pulse width, and magnitude, remain constant during operation (Deuschl et al., 2006; He et al., 2021). Thus, the OL-DBS lacks the capability of adjusting stimulations corresponding to the dynamic symptoms of Parkinsonians. CL-DBS devices, on the other hand, employ a feedback loop to monitor the brain’s clinical condition and adjust stimulation parameters accordingly (Rosin et al., 2011; Little et al., 2013; Wu et al., 2015; Parastarfeizabadi and Kouzani, 2017; He et al., 2021). These adaptive stimulation signals offer multiple significant advantages over OL-DBS system. Firstly, adaptive stimulation signals extend clinical efficacy while reducing side effects (Herron et al., 2016). Recent studies demonstrated that CL-DBS system, with its automatic modification of stimulation parameters, is more effective in reducing PD symptoms compared to OL-DBS system (Rosin et al., 2011). Secondly, the adjustment of stimulation parameters in DBS devices has been found to mitigate or eliminate side effects in a significant proportion of Parkinsonians. (Hamani et al., 2005). Secondly, CL-DBS system utilizes non-continuous stimulation signals, leading to reduced energy requirements of the DBS devices (Herron et al., 2016). Studies reported a 56% reduction in stimulation time and decreased energy demand with CL-DBS system compared to OL-DBS system (Little et al., 2013; Wu et al., 2021). This reduced power requirement may result in fewer neurostimulator battery replacement surgeries. Overall, CL-DBS system offers improved efficiency, fewer surgeries, reduced energy consumption, and extended battery life compared to OL-DBS system. Despite great advantages, several issues are still associated with CL-DBS system.

One challenge is the availability of detectable feedback signals that are stable and robust over time (Hosain et al., 2014). Several electrophysiological biomarkers linked to the symptoms of patients have been introduced for closing the feedback loop including local field potential (LFP), action potential, electroencephalogram potential, and electrocorticogram. The selection of biomarkers for the CL-DBS system in Parkinson’s disease faces several challenges. One of the challenges of selection biomarkers is to understand their evolution over time and their correlation with symptom severity. Studies are required to assess the stability and consistency of biomarkers. Localization specificity is another consideration, requiring biomarkers with good spatial specificity to accurately target specific brain regions. Precise localization and electrode placement are essential for optimal therapeutic outcomes. Robust clinical studies and consensus on selection criteria and assessment protocols are necessary. Addressing these challenges requires collaboration between clinicians, neuroscientists, and engineers to enhance the precision and effectiveness of CL-DBS system, ultimately improving outcomes for individuals with Parkinson’s disease (Rossi et al., 2007).

Another challenge of CL-DBS systems is the design of closed-loop control algorithms for the automatic adjustment of stimulation parameters (Parastarfeizabadi and Kouzani, 2017). A robust control mechanism is essential for CL-DBS systems to enable automatic updates of stimulus settings without the need for manual intervention. Current existing closed-loop controlling algorithms either control one pulse parameter such as amplitude or implement a simple on-off control of stimulations. However, to further optimize the efficiency of the system, it is ideal to set a threshold and continually monitor the biomarker and control stimulation on-off when the signal crosses the threshold. Thus, the development of an optimized controller for programming stimulation parameters is further needed. In addition, CL-DBS devices are expected to consume less power compared with the OL-DBS systems. Nevertheless, CL-DBS devices carry real-time recording and data processing circuits that cause high power consumption for the device. Therefore, there is an urgent need to develop an adaptive CL-DBS system with low power consumption, high intelligence, and minimal side effects to optimize patient outcomes.

Overall, designing CL-DBS algorithms with energy-efficient hardware implementation is essential. Due to the diverse variations in signs and symptoms among Parkinson’s patients, continuous monitoring of PD indicators and making appropriate adjustments to stimulus signals are crucial. Therefore, the development of an intelligent and energy-conscious PD symptom detector and controller is necessary to achieve optimal results for patients while minimizing negative side effects.

### 2.3 Overview of memristive synapse

**Figure 2:**
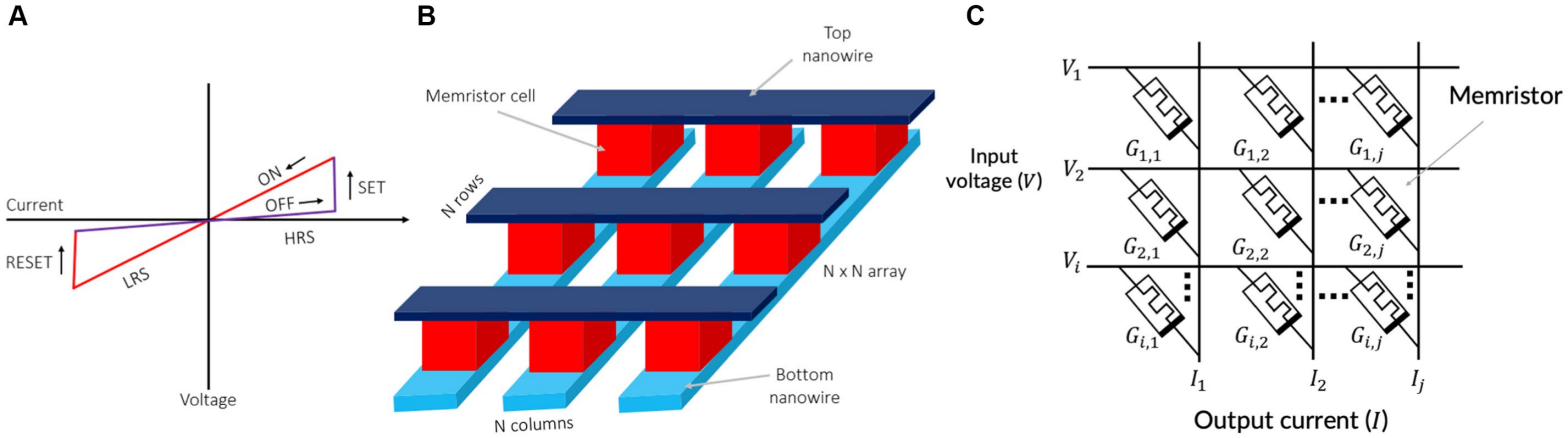
**(A)** Switching characteristics of memristive devices. **(B)** A crossbar array of memristors. **(C)** Vector matrix multiplication using a memristor crossbar.

A memristor is a non-volatile memory device that encodes information into its resistances. Therefore, memristors are also known as resistive random-access memory (ReRAM) or RRAM (Chua, 1971; Strukov et al., 2008; Williams, 2008; Eshraghian et al., 2012; Wong et al., 2012; An et al., 2021a,b; Chua et al., 2022; Zins et al., 2023). The memristor operates by modifying its resistances with a voltage stimuli (Strukov et al., 2008). This property allows memristive devices to exhibit their current–voltage (*I*–*V*) characteristic curves as shown in Figure 2A. Memristors have garnered attention as promising nanodevices for in-memory computing and electronic synapses due to their potential for high-density integration, fast writing and reading times, and high power efficiency (Upadhyay et al., 2019). Importantly, the conductance of a memristor is not solely influenced by the current control signals (applied voltage or current), but also by their history, such as the time integral of charge or flux (Chua, 1971; An et al., 2017). In addition, memristors offer the advantage of being compatible with CMOS (complementary metal-oxide-semiconductor) fabrication processes. This compatibility allows for the seamless vertical integration of memristors with CMOS-based integrated circuits (ICs), forming three-dimensional integrated circuits (3D-ICs).

The typical structure of a memristor is depicted in Figure 2. Memristive devices consist of insulating layers sandwiched between top and bottom nanowire electrodes (Figure 2B). Multiple memristors are commonly fabricated in a crossbar configuration, as illustrated in Figure 2B. This straightforward crossbar structure enables the scaling down of individual memristive devices into approximately 10 nm (Lu et al., 2011).

The crossbar configuration offers a high integration density and random-access capacity (Snider and Williams, 2007). As shown in Figure 2B, with the utilization of the n-rows and n-columns of the crossbar, all *n* × *n* cross points can be accessed. Memristive crossbars also have the capability for analog arithmetic calculations (Min and Corinto, 2021). The characteristics of in-memory computer architecture render it a highly promising approach for advancing neuromorphic systems. Vector-matrix multiplications, widely used in deep learning and in-memory computing, stand to gain significant benefits from this architecture. Figure 2C depicts a memristive vector-matrix multiplication (VMM) engine (Cui and Qiu, 2016), capable of performing analog computations of *I* = *G* · *V* using a conductance matrix G with dimensions *i*-by-*j*.

The VMM engine is composed of two layers of metal wires, denoted as *i* for the input voltage vector and *j* for the output current vector. Each memristor acts as a connection point between the overlapping top and bottom wires. By setting the conductance of the memristor at coordinates *i* on the bottom and *j* on the top to values *G_i,j_*, the output current vector *I* can be generated on the bottom wires when an input voltage vector *V* is applied. The crossbar structure of the memristor allows for sampling the outputs by measuring the accumulated current on each bit line (BL). This facilitates the analog computation of VMM, where input tensors are mapped as voltages loaded in parallel on each word line (WL), and synaptic weights are represented by the conductance of memristor cells in a subarray as:


(1) $$I_{j}=V_{i}G_{i,j}$$

where *V_i_* is the input voltage at *i*-th wordline (WL) and $G_{i,j}$ is the conductance of the memristor cell stacked between *i*-th WL and *j*-th bitline (BL). The crossbar cannot operate properly unless the bottom wires are held at ground potential. Another crossbar and subtraction circuit is required to support negative entries in the conductance (Pino et al., 2012). Memristor crossbars with a high density are able to conduct parallel vector-matrix multiplication while consuming an extremely minimal energy (Zhang et al., 2017). Furthermore, the parameters of the applied voltage pulses can be modulated in order to adapt the memristor’s conductance, offering tremendous potential for the development of adaptive systems with the capacity for online learning (Li and Ang, 2021). Thus, memristors are considered promising nanodevices for electronic synapses in a neuromorphic chips.

## 3 Design of neuromorphic CL-DBS detectors

Neuromorphic systems with memristive synapses are promising next-generation artificial intelligence platforms known for their remarkable energy efficiency. In this paper, we present the development of a novel neuromorphic PD symptom detector for the CL-DBS system. The detector utilizes spiking neural networks (SNNs) to detect and analyze PD symptoms based on spike patterns, particularly in the region of STN. Unlike previous approaches that involve converting spiking signals from the time domain to the frequency domain, our neuromorphic detector directly processes the spiking signals, eliminating the need for time-frequency conversion.

Specifically, the implementation of our neuromorphic PD detector involves the utilization of the long short-term memory (LSTM) architecture. The neural activities used for training are collected using a PD computational model (Kumaravelu et al., 2016). The dataset utilized for training encompasses spike timings spanning from 0 to 2,500 milliseconds per data sample.

To comprehensively evaluate the hardware performance of our detector, a strategic approach is employed. We systematically save the weights and biases during the training process and subsequently integrate them into NeuroSIM as memristive synapses. This integration enables a thorough evaluation, considering both monolithic and heterogeneous 3D chip designs.

**Figure 3:**
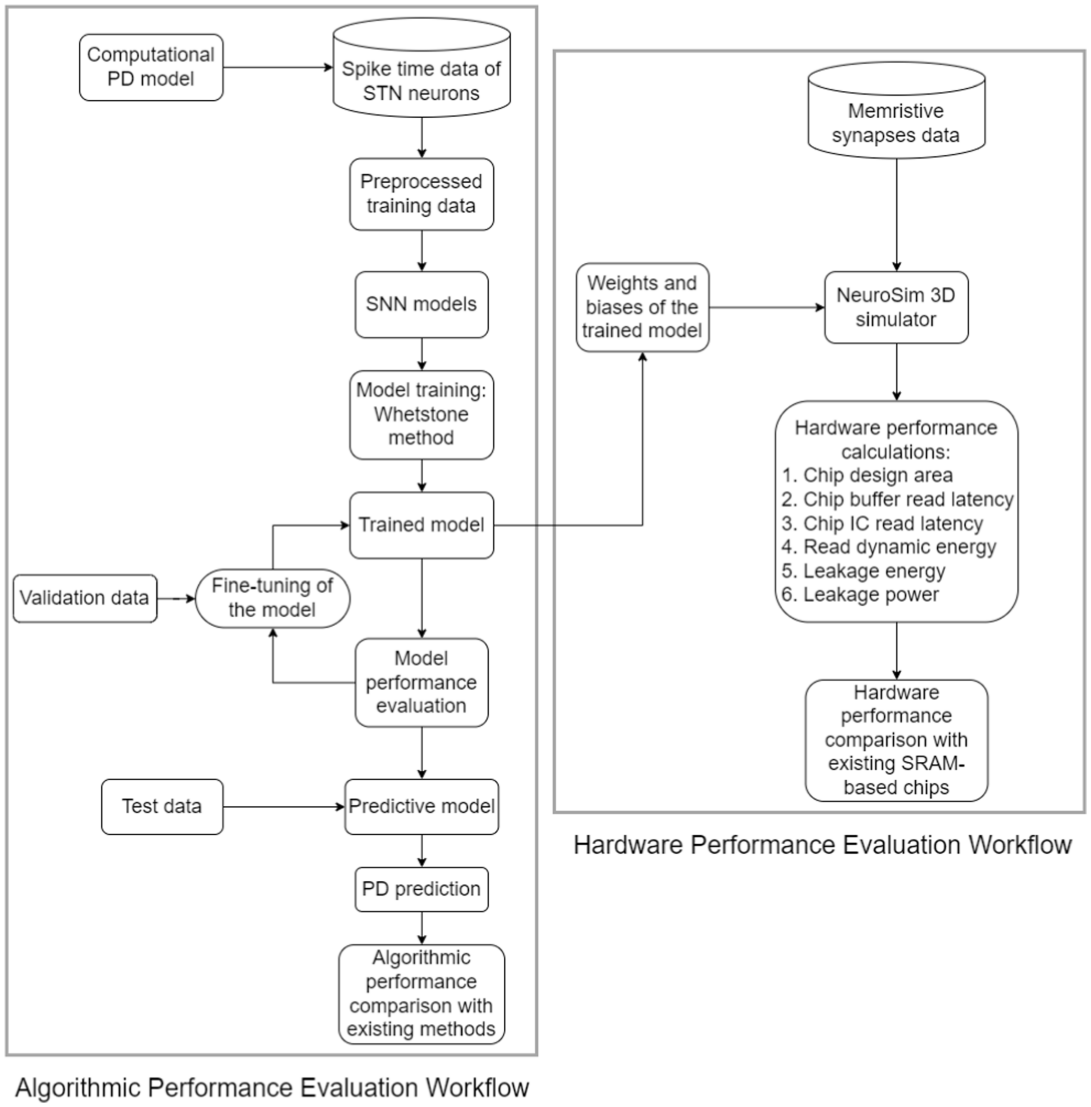
Workflow of the proposed hardware and algorithmic co-design methodology of neuromorphic CL-DBS detectors.

Remarkably, our neuromorphic PD detector exhibits superior performance compared to the conventional 6 T SRAM memory architecture. This superiority is evident in various aspects, including chip design area, latency, and power consumption. The intricacies of the design and assessment methodology are visually depicted in Figure 3, offering a clear illustration of our neuromorphic PD detector’s functionality.

Moreover, a distinct validation dataset is employed as a crucial component of our evaluation process, ensuring a robust assessment of the detector’s performance. The weights and biases derived from the 8-layer detector are meticulously preserved throughout the training phase, and these parameters are seamlessly integrated into NeuroSIM as memristive synapses for a comprehensive analysis of hardware performance.

### 3.1 Acquisition of PD spiking data

**Figure 4:**
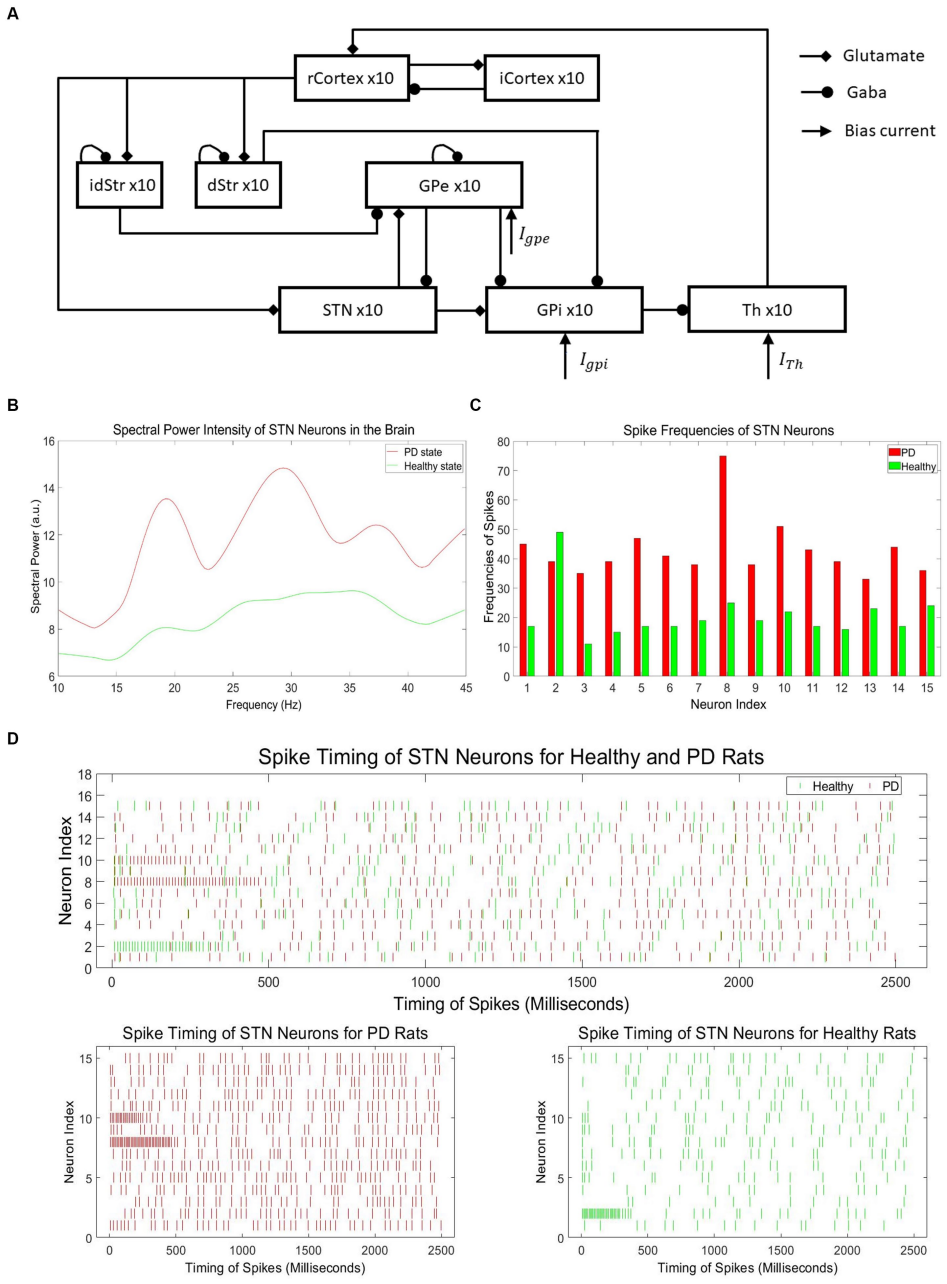
**(A)** Architecture of cortical-basal ganglia-thalamus network PD model (Kumaravelu et al., 2016). **(B)** Spectral power intensity of neurons in the STN region of the brain for PD and healthy states. **(C)** Spike frequencies of neurons in the STN region of the brain for PD and healthy states. **(D)** Neuronal spikes in the STN region for healthy and Parkinson’s disease rats.

The neural activity with typical PD symptoms for training our neuromorphic PD detector is obtained by a computational model, which includes the six brain regions as shown in Figure 4A (Kumaravelu et al., 2016). This model represents the cortical-basal ganglia-thalamus network, incorporating the brain regions of the cortex, striatum, subthalamic nucleus, globus pallidus externa, globus pallidus interna, and thalamus. Each of these regions is modeled using 10 single-compartment neurons. These neurons form a functional network by being interconnected through synapses. The cortex and striatum have stochastic connections, while other regions exhibit structured connections. During simulations, different time steps were tested, and the results remained stable regardless of the time step value.

The network model incorporates various types of connections among the neurons in the six regions. Regular cortex neurons receive excitatory input from thalamic neurons and inhibitory input from randomly selected inhibitory cortex neurons. Inhibitory cortex neurons, on the other hand, receive excitatory input from randomly chosen regular cortex neurons. Direct pathway striatum neurons receive excitatory input from regular cortex neurons and inhibitory input from randomly chosen direct pathway striatum neurons. Indirect pathway striatum neurons receive excitatory input from regular cortex neurons and inhibitory input from randomly selected indirect pathway striatum neurons. Subthalamic nucleus neurons receive inhibitory input from globus pallidus externa (GPe) neurons and excitatory input from regular cortex neurons. Globus pallidus externa neurons receive inhibitory input from any two other globus pallidus externa (GPe) neurons, as well as from all indirect pathway striatum neurons. Globus pallidus interna neurons receive inhibitory input from globus pallidus externa neurons and from all direct pathway striatum neurons. Additionally, some globus pallidus externa and globus pallidus interna neurons also receive excitatory input from subthalamic nucleus neurons. Finally, thalamic neurons receive inhibitory input from globus pallidus interna neurons.

To train our neuromorphic PD detector, we collected spike data from the neurons in the subthalamic nucleus (STN) region of the brain to construct the dataset. According to research on PD, there is an increased power spectrum in low-frequency oscillations in neurons of the basal ganglia (BG) in the Parkinsonian state compared to the healthy state. Hence, the spectral power at low frequencies, specifically beta oscillations (13–30 Hz), can be used as an indicator of PD and healthy states (McConnell et al., 2012). Figure 4B illustrates the intensity of spectral power in representative STN neurons for both PD and healthy states, clearly demonstrating the noticeable difference in beta oscillation levels between the two states. However, conducting spectral analysis on PD spike data to generate beta oscillations is a time-consuming and energy-intensive process. Hence, the utilization of spike timing directly offers notable advantages in terms of temporal and energy efficiency. Considering the difficulties involved in acquiring PD data from experimental studies, we employed a computational PD model (Kumaravelu et al., 2016) to generate a significant volume of spike timing data specifically tailored for PD.

Figure 4C depicts the spike frequencies of representative neurons in the STN region for PD and healthy states, clearly demonstrating the asymmetry between the two states, with significantly higher spike frequencies in the PD state. The PD spike data with no DBS stimuli consists of 1,000 independent samples, each representing the spiking signals of 15 neurons within the range of 0 to 2,500 ms. In total, the dataset contains 1,000 samples, encompassing spike timing information for a total of 15,000 neurons.

The spike timing intervals cover a range of 0 to 2,500 milliseconds, providing detailed temporal information of spikes. Similarly, the healthy spike data with no DBS stimuli comprises 1,000 distinct dataset samples, with each sample containing spike data of 15 neurons within the same 0 to 2,500 milliseconds range. The dataset includes 1,000 samples, capturing spike timing information for a total of 15,000 neurons. Figure 4D shows the spike timing data samples of 15 healthy and PD STN neurons. The spike timing data in the healthy state is much sparser compared to the PD state.

### 3.2 Design and training of spiking long short-term memory for neuromorphic detector

Our neuromorphic detectors can be successfully trained using spike data obtained from the PD biophysical computational model. While SNNs are known for their remarkable energy efficiency, training them using traditional gradient descent techniques becomes challenging due to the non-differentiability of threshold neurons. To address this challenge, a training method called Whetstone (Severa et al., 2019) are employed. The Whetstone approach simplifies hardware implementation by generating binary outputs of “1” or “0” instead of using other complex encoding schemes, such as temporal coding. In the Whetstone training method, the neural networks are initially trained using conventional backpropagation techniques and differentiable activation functions such as the rectified linear unit (ReLU) function. Subsequently, these differentiable activation functions are replaced with non-differentiable threshold functions during training. This transformation of the activation function during the training process is referred to as the sharpening process. Initially, the ReLU function is represented using conventional differentiable functions prior to the training procedure. However, during training, the ReLU function undergoes a transformation into a threshold function. Specifically, a bounded ReLU (bRELU) of an artificial neural network (ANN) gradually evolves into a traditional step function through the utilization of Eq. (2). This modification of the activation function occurs as part of the sharpening process, which enhances the efficiency of data processing and classification.


(2) $$h_{\alpha ,\beta }=\left\{\begin{array}{c} 1,\mathrm{if}\;x_{i}\geq \beta \\ \frac{x_{i}-\alpha }{\beta -\alpha },\mathrm{if}\;\alpha \leq x_{i}\leq \beta \\ 0,\mathrm{if}\;x_{i}\leq \alpha \end{array}\right.$$

The assumption |*β* − 0.5| = |*α* − 0.5| in Eq. (2) illustrates the characteristics of the bounded ReLU (bRELU) activation function. The generic bRELU function, denoted as h_α,β_, undergoes a progressive transformation into a threshold function as α approaches 0.5 and h approaches 1. Importantly, throughout this modification, the activation function remains differentiable, allowing for effective training with gradient descent algorithms. By employing the sharpening procedure, the activation function *h_α,β_* (with *α* = 0 and *h* = 1) is converted into a threshold function. To mitigate potential accuracy loss during training, an adaptive sharpening process can be implemented. This approach periodically evaluates the training accuracy at the end of each epoch and, if the change in training loss is consistently smooth, suspends or terminates the sharpening process. By leveraging threshold neurons, the Whetstone method overcomes the non-differentiability challenge and enables the successful training of SNNs for spike PD symptom detection.

**Figure 5:**
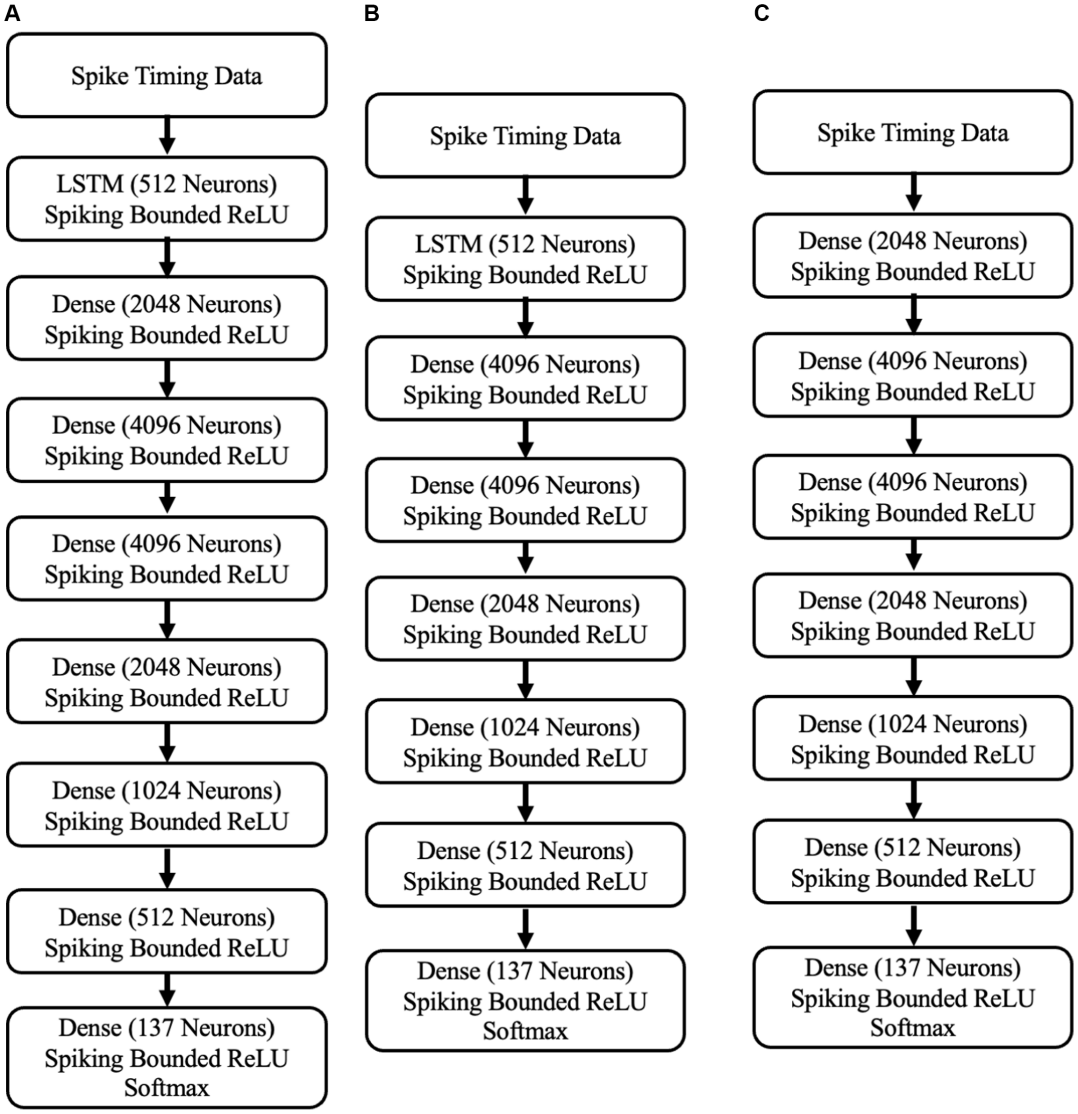
**(A)** The 8-layer neuromorphic PD detector with LSTM. **(B)** A 7-layer neuromorphic PD detector with LSTM. **(C)** the 7-layer neuromorphic PD detector with SNNs.

To detect abnormal neural activities associated with PD symptoms in STN neurons, we have developed three SNN-based neuromorphic PD detectors. These neuromorphic detectors are an 8-layer S-LSTM neuromorphic PD detector, a 7-layer neuromorphic S-LSTM PD detector, and a 7-layer neuromorphic SNN PD detector. The architectures of these detectors are depicted in Figure 5.

To assess their performance in recognizing PD symptoms, these detectors underwent training and validation using 30,000 spike data samples from STN neurons in our computational PD model. The train-test mechanism of SNN algorithms relies on data splitting, where a portion of the data is used for training and the remaining data for evaluation. Therefore, determining the appropriate percentage for training and validation is crucial. In this study, we employed the training-validation data-splitting technique. The data were divided into three groups using different training-validation splits: 60%–40%, 75%–25%, and 90%–10%. Each of the three PD detectors was tested on different data splits to evaluate the performance of the SNN algorithms. All the SNN-based PD detectors were trained for 100 epochs using the adadelta optimizer with a learning rate of 0.05. The classifiers were trained using the Whetstone training method, which incorporates an adaptive sharpening procedure. This procedure gradually transforms a bounded ReLU activation function into a threshold function based on the model’s accuracy and loss performance.

The neuromorphic PD detector was trained and evaluated for 100 epochs using a total of 30,000 spike data samples. The data were divided into three groups using the training (%)—validation (%) split technique. For the 60%–40% split, the training dataset consisted of 18,000 spike timing data samples, while the validation dataset contained 12,000 spike timing data samples. In the case of the 75%–25% split, the training dataset comprised 22,500 spike timing data samples, and the validation dataset had 7,500 spike timing data samples. Lastly, for the 90%–10% split, the training dataset included 27,000 spike timing data samples, and the validation dataset consisted of 3,000 spike timing data samples.

Table 1 presents a comparison of key performance measures for neuormorphic PD detection among the 8-layer S-LSTM, 7-layer S-LSTM, and 7-layer SNN detectors. The comparisons were made using different training-validation data split ratios of 60%–40%, 75%–25%, and 90%–10%.

**Table 1:** Comparison of performance measures of SNN-based PD classifiers on the validation dataset.

Training (%)—validation (%) split ratios	Performance measures	8-layer S-LSTM	7-layer S-LSTM	7-layer SNN
60%–40% splits	ACC	0.9962	0.9960	0.9960
	MCR	0.0038	0.0040	0.0040
	AUC score	0.9998	0.9999	0.9998
	Precision	0.9963	0.9973	0.9960
	Recall/sensitivity	0.9960	0.9947	0.9960
	Specificity	0.9963	0.9973	0.9960
	FPR	0.0037	0.0027	0.0040
	FNR	0.0040	0.0053	0.0040
	F1 score	0.9962	0.9959	0.9960
	MCC	0.9923	0.9921	0.9920
75%–25% splits	ACC	0.9952	0.9949	0.9948
	MCR	0.0048	0.0051	0.0052
	AUC score	0.9998	0.9998	0.9998
	Precision	0.9965	0.9957	0.9960
	Recall/sensitivity	0.9939	0.9941	0.9936
	Specificity	0.9965	0.9957	0.9960
	FPR	0.0035	0.0043	0.0040
	FNR	0.0061	0.0059	0.0064
	F1 score	0.9952	0.9949	0.9948
	MCC	0.9904	0.9899	0.9896
90%–10% splits	ACC	0.9960	0.9960	0.9960
	MCR	0.0040	0.0040	0.0040
	AUC score	0.9997	0.9998	0.9998
	Precision	0.9953	0.9960	0.9967
	Recall/sensitivity	0.9967	0.9960	0.9953
	Specificity	0.9953	0.9960	0.9967
	FPR	0.0047	0.0040	0.0033
	FNR	0.0033	0.0040	0.0047
	F1 score	0.9960	0.9960	0.9959
	MCC	0.9920	0.9920	0.9920

For the 60%–40% data splits, the 8-layer S-LSTM outperforms the 7-layer SNN and the 7-layer S-LSTM in accuracy (ACC), misclassification rate (MCR), recall, false negative rate (FNR), F1 score, and Matthews correlation coefficient (MCC). The 7-layer S-LSTM outperforms the 7-layer SNN and the 8-layer S-LSTM in the area under the ROC curve (AUC score), precision, specificity, and false positive rate (FPR). The 7-layer SNN outperforms the 7-and 8-layer S-LSTM in recall and FNR. Therefore, the 8-layer S-LSTM classifier demonstrates the best performance, while the 7-layer SNN classifier exhibits inferior performance for the 60%–40% splits.

For the 75%–25% data splits, the 8-layer S-LSTM outperforms the 7-layer SNN and the 7-layer S-LSTM in ACC, MCR, precision, specificity, FPR, F1 score, and MCC. The 7-layer S-LSTM outperforms the 7-layer SNN and the 8-layer S-LSTM in recall and FNR. The 7-layer SNN outperforms the 7-and 8-layer S-LSTM in AUC score. Therefore, the 8-layer S-LSTM classifier demonstrates the best performance, while the 7-layer SNN classifier exhibits inferior performance for the 75%–25% splits.

For the 90%–10% data splits, the 8-layer S-LSTM outperforms the 7-layer SNN and the 7-layer S-LSTM in ACC, MCR, recall, FNR, F1 score, and MCC. The 7-layer S-LSTM outperforms the 7-layer SNN and the 8-layer S-LSTM in ACC and MCR. The 7-layer SNN outperforms the 7-and 8-layer S-LSTM in ACC, MCR, AUC score, precision, specificity, FPR, and MCC. Therefore, the 7-layer SNN classifier demonstrates the best performance, while the 7-layer S-LSTM classifier exhibits inferior performance for the 90%–10% splits. These observations indicate the varying performance of the classifiers based on different data split ratios.

**Figure 6:**
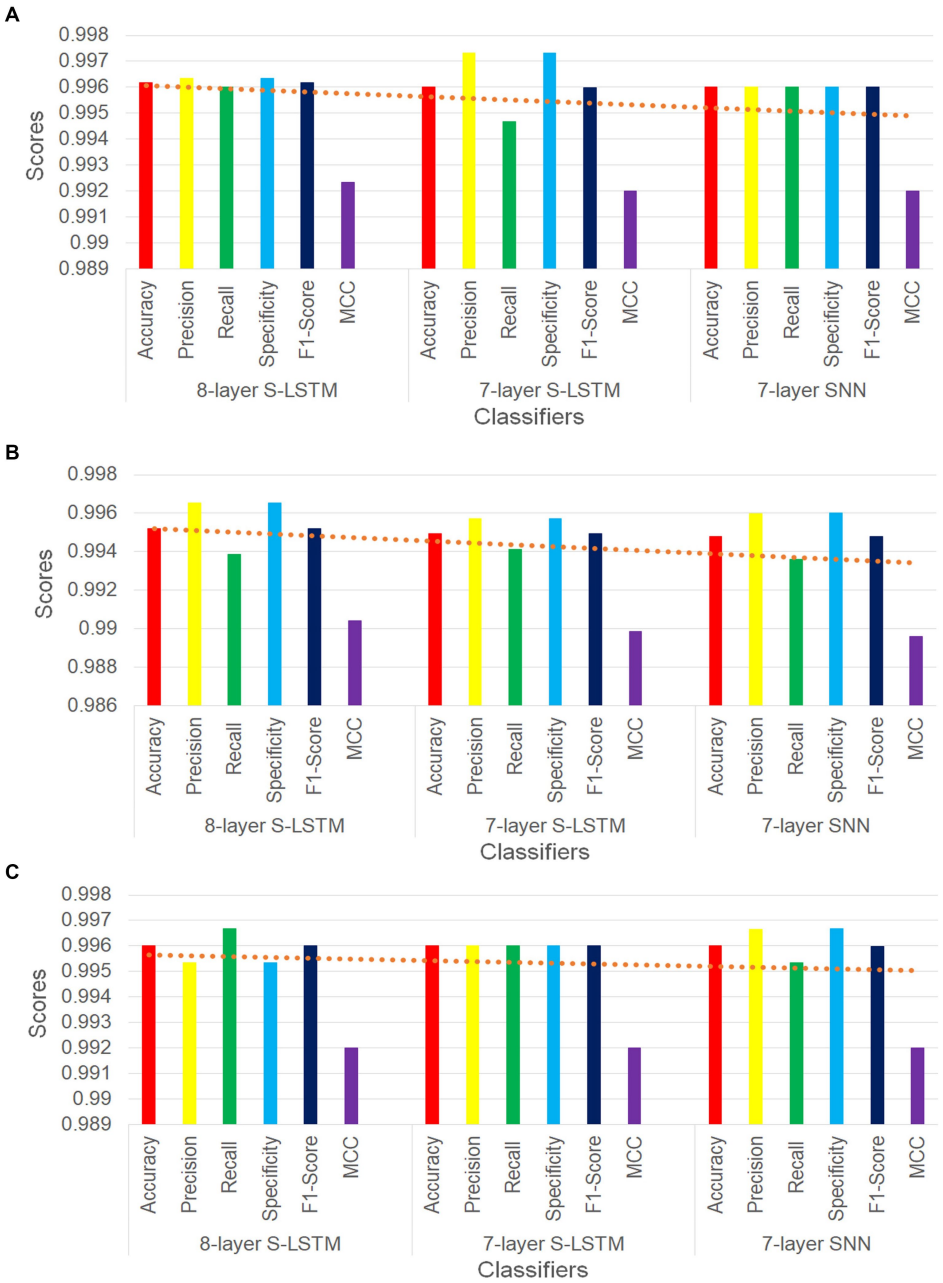
**(A)** Comparison of performance metrics of SNN classifiers for 60%–40% split. **(B)** Comparison of performance metrics of SNN classifiers for 75%–25% split. **(C)** Comparison of performance metrics of SNN classifiers for 90%–10% split.

Figure 6 illustrates six key performance measures for the three PD classifiers with training-validation data split ratios of 60%–40%, 75%–25%, and 90%–10%, respectively. In all three figures, the trend lines consistently demonstrate that the 8-layer S-LSTM classifier outperforms both the 7-layer SNN classifier and the 7-layer S-LSTM classifier. Additionally, the trend lines indicate that the performance of the 7-layer SNN classifier is the lowest among the three classifiers, as all trend lines point downward towards it (see Figure 6).

**Figure 7:**
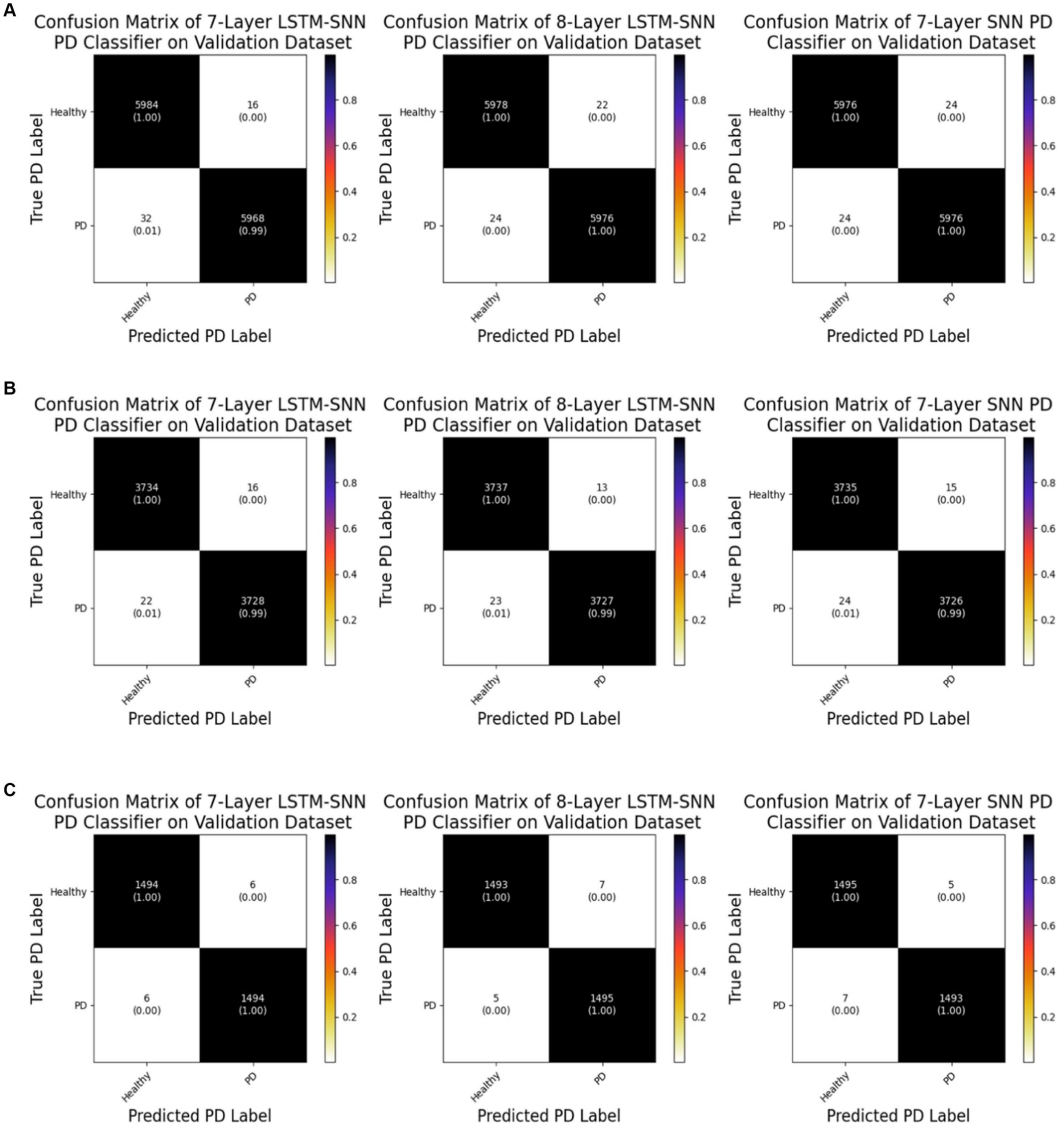
**(A)** Confusion matrices of neuromorphic PD detectors on the validation dataset for 60%–40% split. **(B)** Confusion matrices of neuromorphic PD detectors on the validation dataset for 75%–25% split. **(C)** Confusion matrices of neuromorphic PD detectors on the validation dataset for 90%–10% split.

Figure 7 displays the confusion matrices derived from evaluating the validation dataset using different training validation data splits. These matrices offer insights into the classification performance of the classifiers. For the 7-layer S-LSTM classifier, out of 6,000 healthy samples, 5,984 were correctly labeled as healthy, while 16 were mistakenly labeled as PD. Out of 6,000 PD samples, 5,968 were correctly identified as PD, but 32 were erroneously classified as healthy. For the 8-layer S-LSTM classifier, out of 6,000 healthy samples, 5,978 were accurately classified as healthy, while 22 were misclassified as PD. Regarding the PD samples, 5,976 were correctly labeled as PD, but 24 were incorrectly assigned as healthy. In the case of the 7-layer SNN classifier, out of 6,000 healthy samples, 5,976 were correctly identified as healthy, while 24 were erroneously labeled as PD. Similarly, out of 6,000 PD samples, 5,976 were correctly classified as PD, but 24 were mistakenly categorized as healthy. Based on these observations, it can be concluded that the 8-layer S-LSTM PD classifier exhibits superior performance compared to the other two classifiers, indicating its higher accuracy in classifying PD samples. However, the performance of the other two classifiers, the 7-layer S-LSTM and the 7-layer SNN, could be further improved to enhance their classification accuracy.

Analysis of the confusion matrix reveals that all of the healthy data points were correctly identified, with only one out of the five PD data points being erroneously labeled as healthy. Consequently, we can conclude that our SNN classifier exhibits robustness in accurately identifying PD from new spike timing test data.

### 3.3 Noise robustness analysis of the proposed model

Noise robustness is a critical evolution metric in neuromorphic systems (Yang et al., 2021, 2022a,b; Yang and Chen, 2023; Yang, et al. 2023a,b). To validate the robustness of our model against timing noise in neural signaling, Gaussian noise is introduced to the timing of neural signal firings. Specifically, a series of random numbers drawn from a Gaussian distribution with a specified mean and standard deviation were added to the neural activity timings of the original data samples.

For each non-zero spike timing value in the dataset, we generate a random noise value using Gaussian distribution. This random noise value represents the amount of variability or perturbation that we are adding to the spike timing value. We then combine the original spike timing value with the Gaussian noise value to create a noisy spike timing value that replaces the original value in the dataset at the specified row and column as shown in Eqs. (3) and (4):


(3) $$N=\frac{1}{\sigma \sqrt{2\pi }}e^{-{\left(x-\mu \right)}^{2}/2\sigma^{2}}\text{,}$$

(4) $$x_{i,j}=v_{i,j}+N\text{,}$$

where $x_{i,j}$ is the noisy spike timing value, $v_{i,j}$ is the original spike timing value, and N represents the Gaussian noise with mean ( μ ) and variance ( $\sigma^{2}$ ). This simulates the effect of random noise on the spike timing data, making it more realistic and suitable for the robustness analysis. In specific, the Gaussian noise is added to the neural spike timing dataset using the Algorithm 1.

**Table:** 

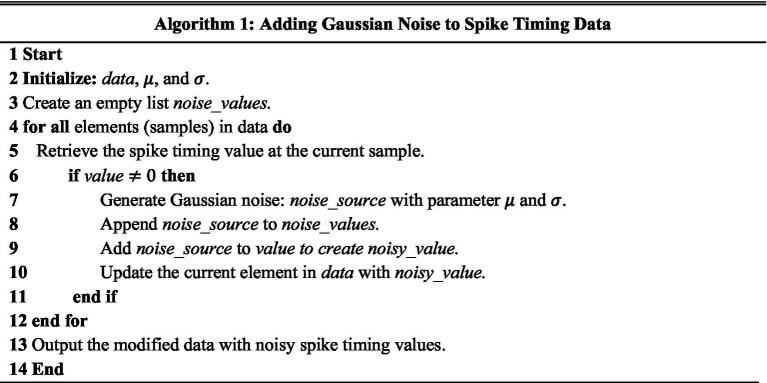

**Figure 8:**
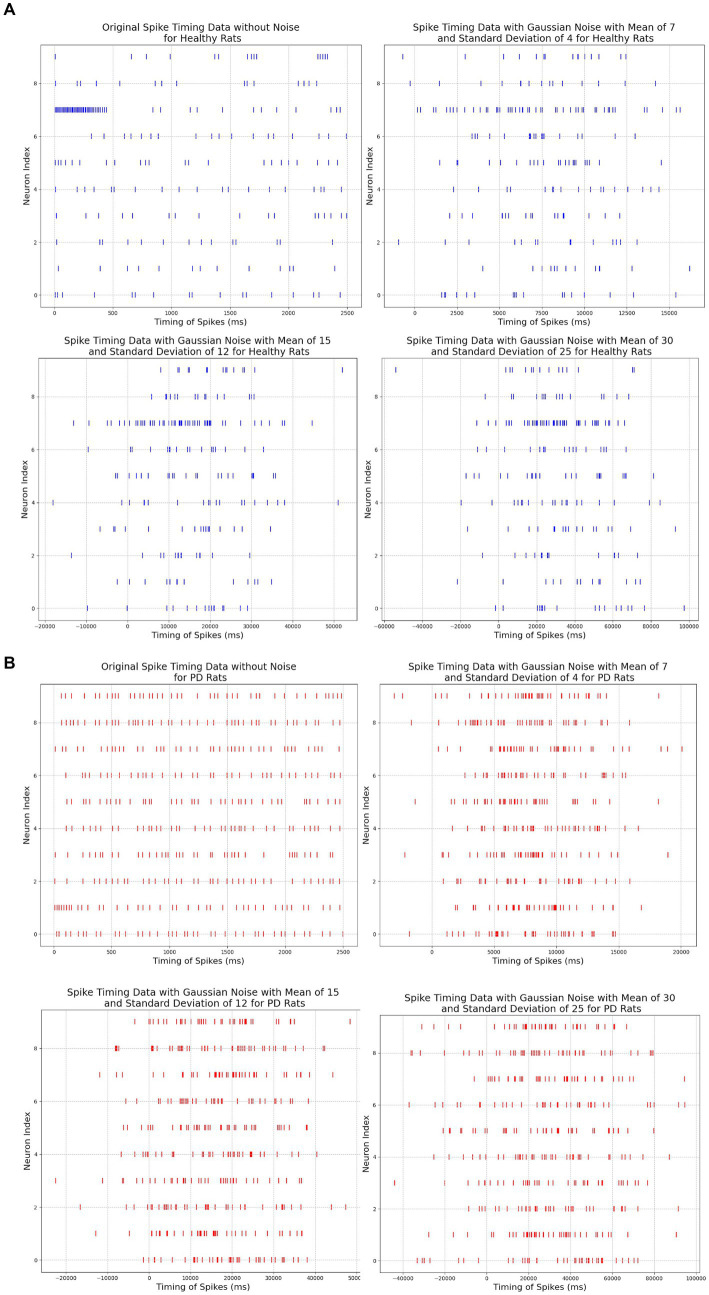
**(A)** Comparison among original and noisy spike timing of healthy rats on three parameter pairs: μ = 7 and σ = 4, μ = 15 and σ = 12, μ = 30 and σ = 25. **(B)** Comparison among original and noisy spike timing of PD rats on three parameter pairs: μ = 15 and σ = 12, μ = 30 and σ = 25, μ = 30 and σ = 25).

To evaluate the robustness of our neuromorphic PD model, we have tested it with three noise settings. In the first noise setting, we applied moderate Gaussian noise with a mean of 7 and a standard deviation of 4. In the second noise setting, we applied moderate to high Gaussian noise with a mean of 15 and a standard deviation of 12, and in the third noise setting, we applied very high Gaussian noise with a mean of 30 and a standard deviation of 25. The spike timing data before and after adding noise signals are illustrated in Figure 8.

**Figure 9:**
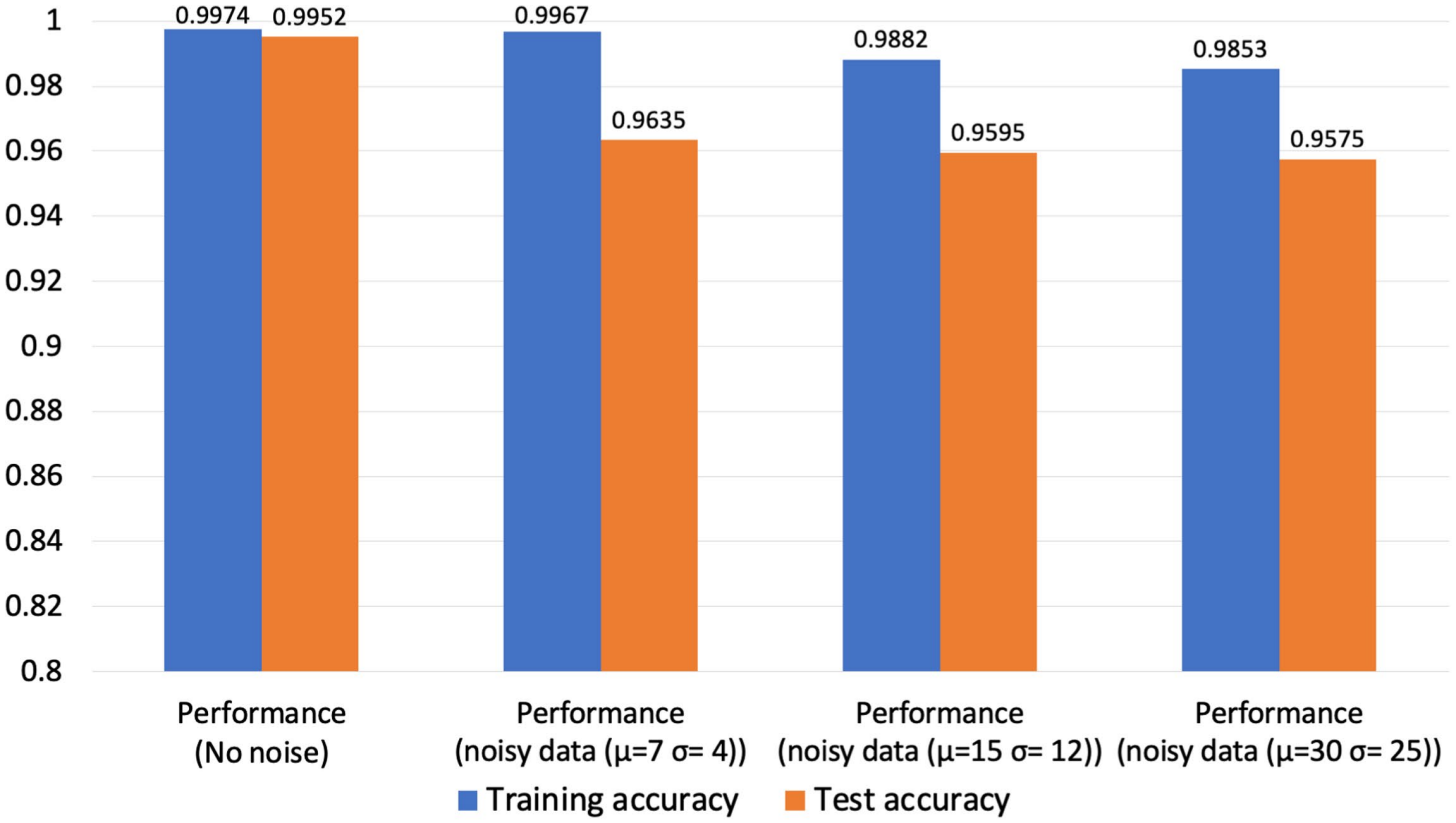
Accuracy trend with increase of noise.

Our neuromorphic PD detector is trained over 100 epochs, employing a dataset partition of 75% for training and 25% for validation to assess the detector’s resilience to noisy data. The dataset comprises a total of 30,000 spike timing samples, with 22,500 samples allocated for the training set and 7,500 for validation. The tabulated results presented in the subsequent table demonstrate the model’s commendable accuracy in detecting Parkinson’s disease from noisy data. Furthermore, we have thoughtfully depicted the confusion matrix for all three cases. As illustrated in Figure 9, although the accuracy of our neuromorphic PD detector decreases with the addition of more intensive noise, the overall accuracy remains at a high level, demonstrating excellent noise immunity capability of our neuromorphic detector.

### 3.4 Hardware performance evaluation and comparison

**Figure 10:**
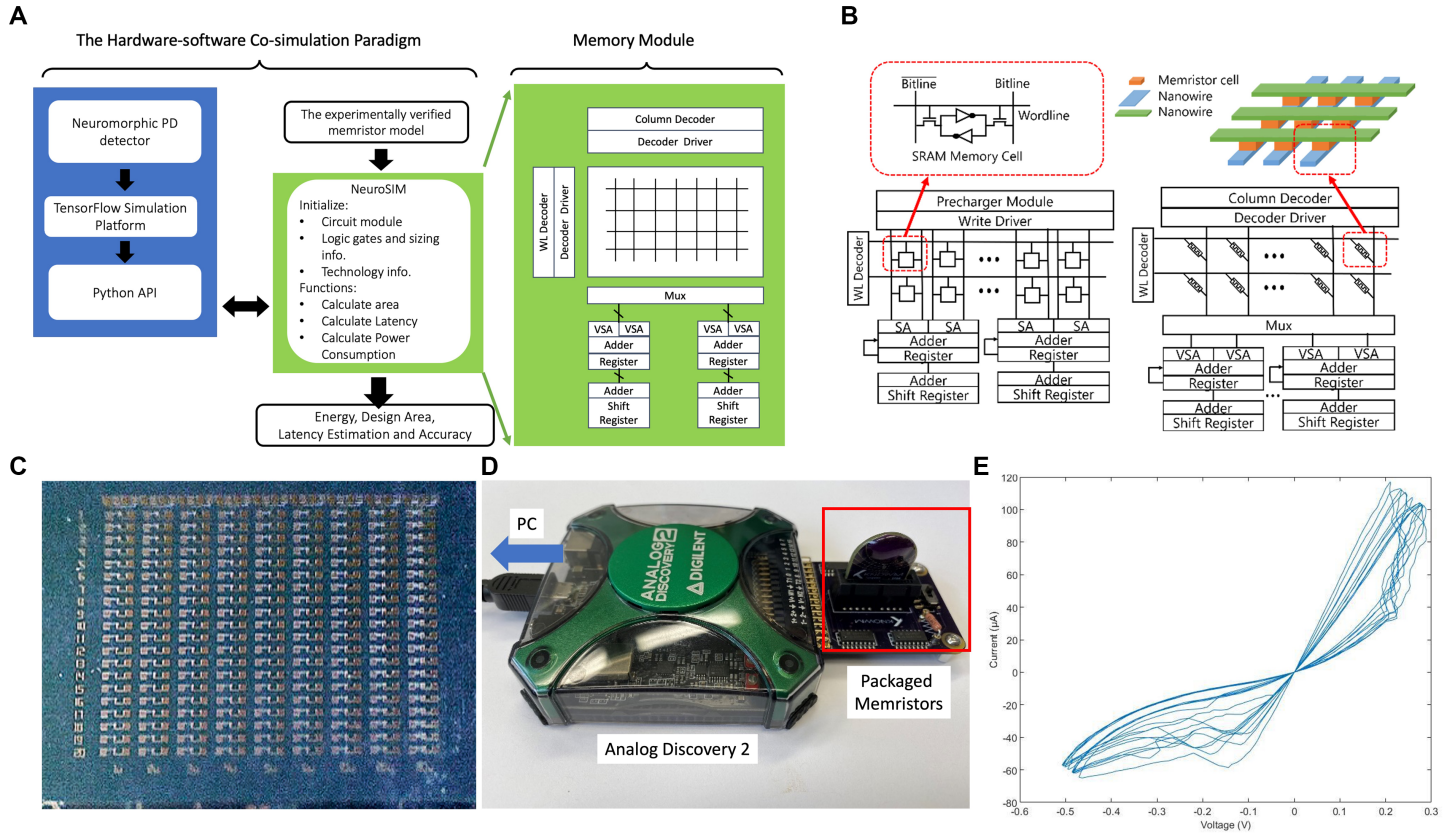
**(A)** Diagram of the hardware-software co-simulation paradigm of our neuormorphic PD detector with NeuroSIM and Whetstone. **(B)** Configuration comparison between the memristive crossbar and the conventional memory array with SRAM as memory cells in NeuroSIM (Chen et al., 2018). **(C)** Raw die of memristors; **(D)** Testing setup of memristors; **(E)** *V*–*I* curve of memristors.

To assess the hardware performance of our neuromorphic PD detector by using memristive synapses, the weights of the neuromorphic PD detector were recorded during training and encoded into the resistance of memristors using a simulator framework named NeuroSim3D (Chen et al., 2018) hardware simulator. Memristors are typically fabricated within a crossbar architecture. As depicted in Figure 10B, nanowires composed of inert cathodes and oxidizable active anodes are situated at the upper and lower regions of the crossbar, respectively. The metallic oxide layer is positioned at the crosspoints where the upper and lower nanowires intersect. This crossbar configuration closely parallels the architecture of a conventional memory array, such as SRAM shown in Figure 10B.

The memristors are added to our neuromorphic PD detector as an electronic synapses (Likharev, 2011; Wang et al., 2012; Bichler et al., 2013; Kornijcuk et al., 2014; Park et al., 2015) storing the weights of neural networks. As the emerging electronic synapses, these memristors replace traditional memory devices, such as SRAM. The parameters of our memristor models are collected by measurement our memristor devices shown in Figures 10C,D. The *V*–*I* curves of the memristors are shown in Figure 10E.

As illustrated in Figure 10B, each memory cell within the memory array is linked to both a wordline and a bitline. The data stored in memristors is encoded in their resistances, and the nanowires serve as the bitline and wordline for accessing the memristive memory cells. Figure 10 illustrates the writing and reading phases of a memristive memory cell. In the writing phase, a voltage pulse, exceeding the set voltage, is applied to the nanowire within the crossbar structure, thus altering the resistance value of the memristor. During the reading stage, the applied voltage is significantly lower than the set voltage to preserve the resistance of the cell unaltered. The resistance value of the selected memristor is calculated as the applied voltage divided by the measured current at the end of the nanowire. The weight matrices are mapped onto the passive memristor crossbar using memory cell selection devices.

Figure 10B shows a traditional SRAM. NeuroSIM conducts weight sum and update operations in a row-by-row fashion (Chen et al., 2018). Row selection is activated through the WL decoder, and the BLs are precharged for each cell access. Memory data is captured by the sense amplifier (S/A). Subsequently, the adder and register are employed to sum the weight values in a row-by-row manner. By substituting SRAM core memory with memristors, the architecture remains largely unaltered shown in Figure 10B. The weighted sum operation in the memristor-based synaptic core also follows a row-by-row style, with the incorporation of multiplexers (MUX) (Chen et al., 2018).

To assess the performance of our neuromorphic PD detector, encompassing design area, latency, and energy efficiency, we have established a hardware-software co-simulation using NeuroSIM (Chen et al., 2018), as depicted in Figure 10A. The model is constructed through the following steps:

Firstly, our neuromorphic PD detector is built of multiple layers of S-LSTM for detecting power density at the beta bandwidth of the STN region. During the training progress, the weights and neural network configuration of the S-LSTM are monitored and stored.

Secondly, our experimentally verified memristor model is incorporated into the micro-architecture simulator *NeuroSIM* (Chen et al., 2018) incorporating parameters such as on-state resistance, off-state resistance, and others. The deployment method assesses the neural network’s performance within an offline training environment, which necessitates local computation. In contrast to online learning, offline learning training maintains the trained neural network on the client side, handling all prediction computations locally (Lane et al., 2015), due to the constraints imposed by limited power and space budgets energy.

Finally, the performance improvements of our memristor on energy efficiency, design area, and execution latency are estimated through the co-simulation paradigm. The pseudocode of our hardware-software co-simulation paradigm is introduced in Figure 10 and in Algorithm 2.

**Table:** 

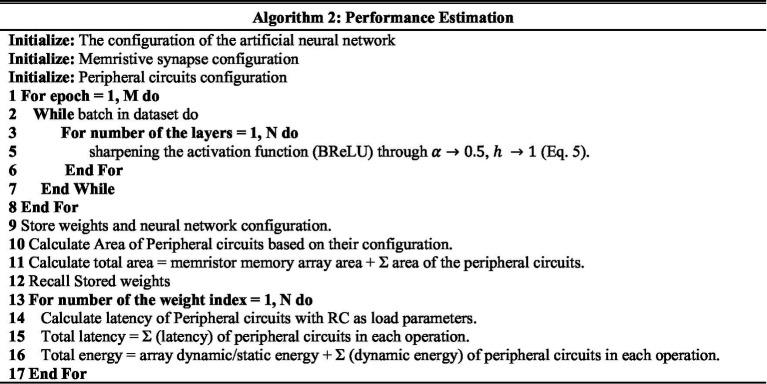

Table 2 shows the values used to set the simulation parameters of the 3D NeuroSim simulator for both monolithic and heterogeneous 3D memristor and SRAM chip designs.

**Table 2:** Settings of simulation parameter values of the NeuroSim3D hardware simulator.

Type of 3D	Monolithic 3D			Heterogeneous 3D		
Device	SRAM	Memristor 1	Memristor 2	SRAM	Memristor 1	Memristor 2
Clock frequency (GHz)	1	1	1	1	1	1
Chip operation temperature (K)	311	311	311	311	311	311
Activation neuron	ReLU	ReLU	ReLU	ReLU	ReLU	ReLU
Technology	22	22	22	22	22	22
Feature size/feature size top (nm)	40	40	40	40	40	40
Device precision	2	2	2	2	2	2
Subarray size	128 × 128	128 × 128	128 × 128	128 × 128	128 × 128	128 × 128
Read voltage (V)	1.1	0.5	0.5	1.1	0.5	0.5
Read pulse width (ns)	N/A	10	10	N/A	10	10
Wire width	40	40	40	40	40	40
Structure	6 T	1T1R	1T1R	6 T	1T1R	1T1R
*R*_ON_	N/A	6e3	12e3	N/A	6e3	12e3
*R*_OFF_	N/A	6e3 × 150	12e3 × 150	N/A	6e3 × 150	12e3 × 150

**Figure 11:**
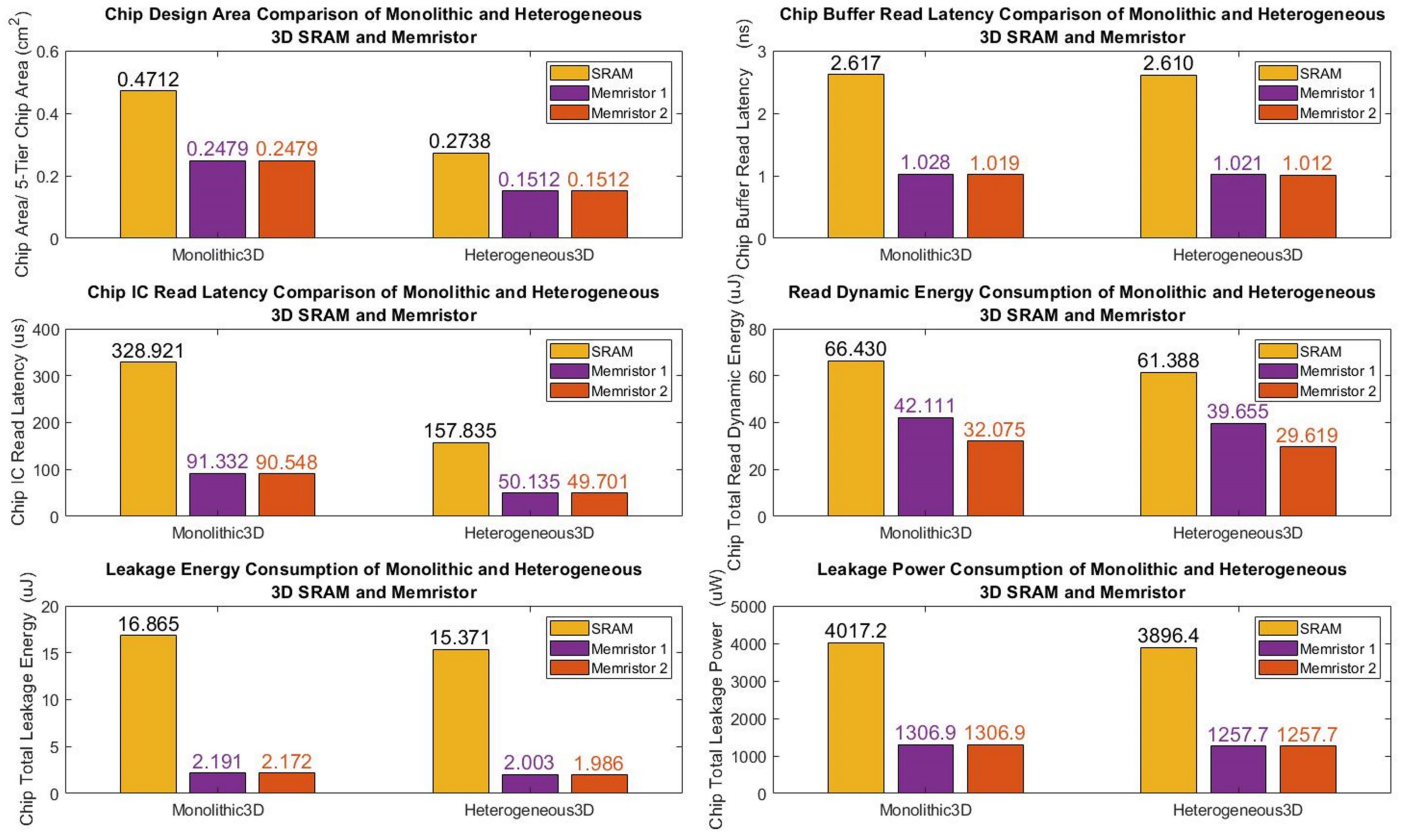
Performance comparison of monolithic and heterogeneous 3D SRAM and memristor hardware.

Figure 11 illustrates our designs offer a significant reduction in the chip design area, with a 47.5% decrease for monolithic 3D and 44.8% for heterogeneous 3D, when compared to conventional SRAM-based designs (see Table 3).

**Table 3:** Breakdown of hardware performance of monolithic and heterogeneous 3D SRAM and memristive chips.

Type of 3D	Monolithic 3D			Heterogeneous 3D		
Device	SRAM	Memristor 1	Memristor 2	SRAM	Memristor 1	Memristor 2
Chip area	4.71e + 07 μm^2^	2.48e + 07 μm^2^	2.48e + 07 μm^2^	2.74e + 07 μm^2^	1.51e + 07 μm^2^	1.51e + 07 μm^2^
Chip clock	5.17 ns	2.10 ns	2.07 ns	5.15 ns	2.08 ns	2.06 ns
Chip layer-by-layer read latency	3.39e + 06 ns	1.32e + 06 ns	1.31e + 06 ns	3.19e + 06 ns	1.24e + 06 ns	1.23e + 06 ns
Chip total read dynamic energy	6.64e + 07 pJ	4.21e + 07 pJ	3.21e + 07 pJ	6.14e + 07 pJ	3.97e + 07 pJ	2.96e + 07 pJ
Chip total leakage energy	1.69e + 07 pJ	2.19e + 06 pJ	2.17e + 06 pJ	1.54e + 07 pJ	2.00e + 06 pJ	1.99e + 06 pJ
Chip total leakage power	4017.15 μW	1306.87 μW	1306.87 μW	3896.45 μW	1257.73 μW	1257.73 μW
Chip buffer read latency	2.62e + 06 ns	1.03e + 06 ns	1.02e + 06 ns	2.61e + 06 ns	1.02e + 06 ns	1.01e + 06 ns
Chip buffer read dynamic energy	713,257 pJ	465,134 pJ	465,134 pJ	234,315 pJ	152,781 pJ	152,781 pJ
Chip read latency	328,921 ns	91332.2 ns	90547.7 ns	157,835 ns	50134.6 ns	49,701 ns
Chip IC read dynamic energy	7.55e + 06 pJ	4.53e + 06 pJ	4.53e + 06 pJ	3.09e + 06 pJ	2.48e + 06 pJ	2.48e + 06 pJ
Energy efficiency TOPS/W	12.122	22.7915	29.483	13.1542	24.2376	31.9473
Throughput TOPS	0.362874	0.9355	0.943605	0.386731	0.990615	0.999258
Throughput FPS	294.58	759.436	766.016	313.947	804.178	811.194
Compute efficiency TOPS/mm^2^	0.00770103	0.0377346	0.0380615	0.0141229	0.065512	0.0660836
Power density (W/mm^2^)	0.000635296	0.00165564	0.00129097	0.00107364	0.0027029	0.00206852

Additionally, the first monolithic and heterogeneous 3D memristive designs demonstrate a substantial reduction in chip buffer read latency, with a decrease of 60.7% and 60.9% respectively, compared to SRAM architecture. Similarly, the second monolithic and heterogeneous 3D memristive designs achieve a reduction in chip buffer read latency of 61.06% and 61.2% respectively, compared to SRAM architecture. Furthermore, the first monolithic and heterogeneous 3D memristive designs significantly decrease chip read latency by 72.2% and 68.3% respectively, compared to SRAM architecture. Similarly, the second monolithic and heterogeneous 3D memristive designs result in a reduction of read latency by 72.5% and 68.5% respectively, compared to SRAM architecture. Furthermore, the first neuromorphic memristive circuit demonstrates a lower read dynamic energy consumption, with a reduction of 36.6% for monolithic 3D architectures and 35.3% for heterogeneous 3D systems compared to conventional 6 T SRAM. The second neuromorphic memristive circuit achieves an even greater reduction in read dynamic energy consumption, with decreases of 51.7% for monolithic 3D architectures and 51.8% for heterogeneous 3D systems compared to conventional 6 T SRAM.

Moreover, the first monolithic and heterogeneous 3D neuromorphic-based memristive architectures exhibit a substantial reduction in leakage energy consumption, with decreases of 86.180% and 87% respectively, compared to traditional 6 T SRAM. Similarly, the second monolithic and heterogeneous 3D neuromorphic-based memristive architectures demonstrate lower leakage energy consumption, with reductions of 87.1% and 87% respectively, compared to conventional 6 T SRAM.

Finally, when compared to SRAM-based chip designs, both monolithic and heterogeneous 3D memristive architectures show a significant decrease in leakage power consumption, with reductions of 67.5 and 67.7%, respectively.

Comparing the performance of these 7-layer SNN spike PD detectors with the beta oscillation detector by Kerman et al. (2022) is a valuable approach to evaluating the effectiveness of the detector. To conduct a fair comparison, we created a separate test dataset using spike timing data from 10 neurons in the STN region of the brain. This test dataset intentionally had a different spike timing data range, spanning from 0 to 2,000 milliseconds, compared to the training and validation datasets.

Table 4 summarizes the comparison of our work with other state-of-the-art CL-DBS systems. From Table 4, we can conclude that our 3D neuromorphic PD detector has outperformed in terms of recognition accuracy, showing an increase of 7.3% and 25%. Furthermore, it has significantly reduced the chip design area by 99.95% and 90.52%.

**Table 4:** Comparison of spike time PD classifier performance with beta oscillation detector performance.

Evaluation metrics	PD detector (Kerman et al., 2022)	CL-DBS system (Gao et al., 2020)	This work
Signal domain	Frequency domain	Frequency domain	Time domain
Hardware	2D memristive neuromorphic system	FPGA	3D memristive neuromorphic system
Chip area	1.69e + 08 μm^2^	N/A	1.51e + 07 μm^2^
Chip energy	9.13e + 7 nJ	N/A	39.27 nJ
Model/algorithm	Spiking MLP	Reinforcement learning	Spiking LSTM
Training accuracy	0.93	N/A	0.9977
Validation accuracy	N/A	N/A	0.9948
Inference accuracy	0.715	N/A	0.90

## 4 Future research

This study presents a design of memristor-based neuromorphic PD detector for CL-DBS system. Nonetheless, the PD detector alone cannot constitute a comprehensive CL-DBS system. An intelligent control mechanism within the feedback loop, as illustrated in Figure 1B, stands as a critical component in a neuromorphic CL-DBS system. In the future, we intend to design and analyze a neuromorphic controller for the CL-DBS system. This neuromorphic controller will also be built upon memristor systems and spiking neural networks.

Another potential research direction involves the utilization of off-the-shelf neuromorphic chips, such as Intel Loihi chip (Davies et al., 2021), for the evaluation and validation of our neuromorphic PD detector and controller. Neuromorphic chips present an emerging and energy-efficient hardware for artificial intelligence (Severa et al., 2019). The Intel Loihi neuromorphic chips employ a digital-analog mixed design, enabling adaptive self-modifying event-driven fine-grained parallel computations. Impressively, these chips achieve exceptional energy efficiency, with less than 81 pJ per neuron update and less than 24 pJ per synaptic operation when operating at 0.75 V. This translates into a substantial reduction in energy usage, surpassing traditional GPUs (graphics processing units) by a factor of 109 and outperforming CPUs (central processing units) by a factor of 23 (Schuman et al., 2017; Blouw et al., 2019; Roy et al., 2019). Notably, one of the latest neuromorphic chips, DYNAPs, has been applied to processing EMG signals with remarkably low power consumption, as little as 614 μW (Sharifshazileh et al., 2021). In the future, the neuromorphic PD detector and controller, implemented with neuromorphic chips, will be incorporated into our PD animal models for real-time testing.

Lastly, we intend to design and fabricate our own neuromorphic chips to further enhance the energy efficiency and intelligence of the CL-DBS system. This project encompasses the design of electronic neurons and synapses using application-specific integrated circuits (ASICs) and memristors. Within this project, we will assess our design using a computational model of Parkinson’s disease (PD) (Davie, 2008; Jankovic, 2008; Ghasemi et al., 2018; Zhou et al., 2018; Lozano et al., 2019; Su et al., 2019). The PD model will provide brain neural activities as input for our circuit design.

Furthermore, our design is set to move forward to the tape-out stage. In this phase, we plan to develop a straightforward neuromorphic chip using electronic neurons and memristive synapses, specifically tailored for CL-DBS systems. Memristors will be integrated into our neuromorphic chip as electronic synapses to further enhance energy efficiency. If successful, this project’s outcome will yield more intelligent and energy-efficient implanted/wearable medical devices for CL-DBS systems. The resulting techniques will also have a broader impact on the future development of wearable and implanted medical devices by significantly reducing their size, weight, energy consumption, and, most importantly, making them more adaptive and intelligent.

## 5 Conclusion

In this paper, we have presented a novel neuromorphic PD detector for CL-DBS utilizing S-LSTMs and memristive synapses. To the best of our knowledge, this is the first technique that integrates memristors and S-LSTMs into the CL-DBS system for spike-time-based PD detection. The proposed neuromorphic-based memristive design chip outperforms conventional SRAM-based architecture, showing significant improvements in chip area, latency, energy usage, and power consumption. In the case of monolithic 3D architecture, the chip achieves a reduction of 47.4% in chip area, 66.63% in latency, 65.6% in energy usage, and 67.5% in power consumption. Similarly, for heterogeneous 3D architecture, the chip exhibits reductions of 44.8% in chip area, 64.75% in latency, 65.28% in energy usage, and 67.7% in power consumption. These advancements in chip design hold tremendous promise for the future development of implanted CL-DBS devices.

## Data availability statement

The raw data supporting the conclusions of this article will be made available by the authors upon request, without undue reservation.

## Author contributions

MS: Data curation, Formal analysis, Software, Writing – original draft, Writing – review & editing. YZ: Formal analysis, Funding acquisition, Project administration, Writing – review & editing. HA: Conceptualization, Funding acquisition, Investigation, Project administration, Resources, Supervision, Writing – original draft, Writing – review & editing.
